# Single-Chain Variable Fragment Antibody of Vascular Cell Adhesion Molecule 1 as a Molecular Imaging Probe for Colitis Model Rabbit Investigation

**DOI:** 10.1155/2019/2783519

**Published:** 2019-01-20

**Authors:** Chunbao Liu, Jun Zhou, Xiaojie Cheng, Liang Xia, Junfen Zhou, Shufang Xu, Yichun Wang, Yongxue Zhang, Diyu Lu

**Affiliations:** ^1^Department of Nuclear Medicine, The Central Hospital of Wuhan, Tongji Medical College, Huazhong University of Science and Technology, Wuhan 430014, China; ^2^Key Laboratory for Molecular Diagnosis of Hubei, The Central Hospital of Wuhan, Tongji Medical College, Huazhong University of Science and Technology, Wuhan 430014, China; ^3^Department of Nuclear Medicine, Union Hospital, Tongji Medical College, Huazhong University of Science and Technology, Wuhan 430022, China; ^4^Hubei Province Key Laboratory of Molecular Imaging, Union Hospital, Tongji Medical College, Huazhong University of Science and Technology, Wuhan 430022, China

## Abstract

Vascular cell adhesion molecule-1 (VCAM-1) can be a promising target for colitis study because of its critical role in inflammation development. Single-chain variable fragment (scFv) antibody presents fast blood clearance when served as an imaging probe. We applied the probe of ^99m^Tc-scFv-VCAM-1 to colitis rabbit to examine its imaging performance. The colitis model rabbit was prepared, and a typical inflammatory lesion was confirmed in the colon. The probe of ^99m^Tc-scFv-VCAM-1 was synthesized and injected into the model animal before imaging examination. Scintigraphy detected colitis lesions in both SPECT planar and SPECT/CT fused images, with higher target-to-nontarget ratios in the model group (2.71 ± 0.31) than those in the control group (1.12 ± 0.10). Biodistribution study determined tracer uptake in different organs, and autoradiography (ARG) confirmed probe accumulation in colon lesions. The uptake ratio of the model colon to the control colon was 4.71 ± 0.61 in quantitative analysis of the ARG regions of interest. Stronger VCAM-1 expression in the model colon than that in the control colon was confirmed by western blotting and immunohistochemistry. Our imaging study indicates molecular imaging with scFv-VCAM-1 as a promising way for inflammatory bowel disease diagnosis and evaluation.

## 1. Introduction

Inflammatory bowel disease (IBD), including ulcerative colitis and Crohn's disease, is a chronic idiopathic disease that emerges as a common global health burden with rising prevalence [1]. In spite of its unclear etiology, different theories have been making efforts to explain the pathogenesis. Recent findings believe that environmental, genetic, and immune factors work together to induce abnormal immune response to enterointestinal antigens [2], leading to inflammatory mediators generation, leukocytes infiltration, and impairment in intestinal epithelial barrier [3].

The evaluation method of IBD lesion development and inflammation activity, such as endoscopy and ultrasonography, guides therapeutic strategy and indicates prognosis [4]. However, to develop a precise monitoring method with an excellent diagnostic property and noninvasive feature remains to be challenging [5]. Molecular imaging is an ideal strategy to noninvasively visualize disease occurrence and development on the biochemical and molecular biological level [6]. Thus, molecular imaging may be a promising way to serve as a complementary method to endoscopy for IBD severity surveillance.

Vascular cell adhesion molecule-1 (VCAM-1) promotes the adhesion and recruitment of inflammatory cells to lesions by mediating the firm adhesion of leucocytes to endothelial cells, making it a critical role in inflammation development [7]. So VCAM-1 can be an eligible target for IBD molecular imaging study. Researchers have used anti-VCAM-1 monoclonal antibody as a scintigraphy tracer to evaluate rat colitis [8], and higher radioactivity uptake was observed in the colon of the colitis rats than that of the control animals. Another study coated mesenchymal stem cells with anti-VCAM-1 antibody to enhance their delivery to the colon and increase the therapeutic effectiveness [9]. But intact monoclonal antibody (150 kDa) usually presents poor tissue penetration and slow blood clearance when employed as an imaging tracer [10], and it takes several days to reach a satisfactory target-to-background (T/B) ratio. The development of engineered antibody technique brings small-sized antibody available [11], such as Fab (50–55 kDa), single-chain variable fragment (scFv, 28 kDa), nanobody (15 kDa), and affibody (7 kDa) [12]. Our previous work has labeled anti-VCAM-1 scFv with fluorescent dye and radionuclide to synthesize imaging probes for atherosclerosis detection [13], and these probes demonstrate excellent imaging properties in both mouse and rabbit atherosclerotic lesions. We believe that the imaging probe of anti-VCAM-1 scFv may also work well in the typical inflammatory lesion of colitis.

Our purpose in this study was to reveal the colon inflammation activity in colitis model rabbits with the scintigraphy probe of ^99m^Tc-labeled scFv-VCAM-1. Our hypothesis is that the imaging method can detect colitis lesion in a short probe circulation time. The imaging performance is examined by single-photon emission computed tomography (SPECT), biodistribution, and autoradiography.

## 2. Materials and Methods

### 2.1. Imaging Probe Preparation

Our previous study had prepared scFv targeting VCAM-1 (scFv-VCAM-1) by the phage display method (Shanghai Raygene Biotech Company, Shanghai, China) and tested its binding affinity by enzyme-linked immunosorbent assay (ELISA) to confirm the reactivity with rabbit VCAM-1 antigen [13]. According to the previously described procedure, we used succinimidyl 6-hydraziniumnicotinate hydrochloride (SHNH, Solulink, Inc., San Diego, CA, USA) as the bifunctional chelator to link ^99m^Tc to scFv-VCAM-1 [13]. The imaging probe of ^99m^Tc-scFv-VCAM-1 was successfully acquired. The labeling yield, the radiochemical purity, and the stability were determined by instant thin layer chromatography (ITLC). The in vitro binding assay of ^99m^Tc-scFv-VCAM-1 with VCAM-1-positive and VCAM-1-negative cells was expressed in another work [14].

### 2.2. Colitis Rabbit Model Preparation and Identification

**Figure 1:**
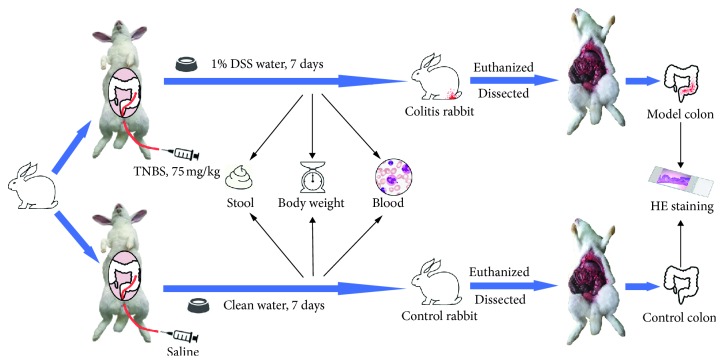
Flow chart of the rabbit colitis induction and the characteristics identification.

Our animal studies were approved for animal welfare by the Institutional Animal Care and Use Committee of Tongji Medical College, Huazhong University of Science and Technology. Male New Zealand white rabbits aged 4 months with a weight of 2–2.5 kg were provided by Animal Center of Tongji Medical College (Huazhong University of Science and Technology, Wuhan, China) and randomly distributed into the model group and the control group. Dextran sulfate sodium (DSS; Sigma-Aldrich, St Louis, MO, USA) and 2,4,6-trinitrobenzenesulfonic acid (TNBS; J&K Chemical Ltd., Shanghai, China) were used to induce colitis lesions in the model group according to other reports [15] (Figure 1). In brief, after being anesthetized with 30 mg/kg dosage of 3% pentobarbital sodium, the rabbits were inserted with a lubricated polyethylene catheter into the colon for about 15 cm proximal to the anus. Each model rabbit was instilled 75 mg/kg of TNBS (dissolved in 50% ethanol, 10 ml of administration volume) into the lumen of the colon through the catheter. As a control, 10 ml of saline was administered in the same way. Then, the rabbits were housed in thermoregulated and humidity-controlled rooms with free access to standard laboratory diet. The model rabbits were fed with ad libitum DSS water (concentration of 1 g/100 ml, dissolved in clean water), whereas the control group received clean water. Blood, stool, and body weight of both the model and control rabbits were checked every day. At day 7 of colitis induction, the rabbits were euthanized with an overdose of pentobarbital sodium, and colon hematoxylin-eosin (HE) staining was performed to confirm lesion existence.

### 2.3. Animal SPECT Imaging

After 12 hours of fasting, 5 model rabbits and 5 control rabbits were injected 74 MBq of ^99m^Tc-scFv-VCAM-1 via marginal ear vein. Then, the rabbits were anesthetized with 30 mg/kg dosage of 3% pentobarbital sodium and placed in the prone position on the scanner bed of a SPECT/CT (Symbia T6, Siemens, Erlangen, Germany) at 3 h postinjection to acquire planar images with the SPECT detector situated on the rabbit ventral side. Each acquisition required 4 min, and the matrix was 256 × 256 pixels with low-energy high-resolution collimation. The target-to-nontarget activity ratio (T/NT) was determined by setting the region of interest (ROI) covering the distal colon as the target region and the contralateral area as the nontarget region. The blocking imaging was also taken to determine the specificity of the imaging probe. The model rabbits were administered a 50-fold dose of unlabeled scFv-VCAM-1 (50 nmol) 1 h prior to the injection of the radioactive probe. Then, the images were acquired in the same way.

Subsequent tomography images were acquired to confirm abdominal radioactivity accumulation in planar images. In brief, 10 ml of 50% diluted iopamidol (Bracco Sine Pharmaceutical Corp. Ltd., Shanghai, China) was instilled into the lumen of the colon via a lubricated polyethylene catheter that was inserted into the rabbit's anus. Then, SPECT tomography images were acquired by two parallel detectors rotating around the rabbit for 32 projections. The acquisition was 10 s per projection, with a matrix of 128 × 128 pixels focusing on the abdominal region. The abdominal computed tomography (CT) scan (100 mA, 130 kV, 1 mm slice thickness) was also acquired for SPECT/CT fusion imaging.

### 2.4. Biodistribution Study

After SPECT planar and tomography imaging, the rabbits were sacrificed for biodistribution study with an overdose injection of pentobarbital sodium. Samples of interest (blood, brain, myocardium, lung, liver, spleen, kidney, stomach, intestine, colon, muscle, and bone) were harvested, rinsed, and weighed. An automatic well-type gamma counter (WIZARD2 2470, Perkin-Elmer, Norwalk, CT, USA) was used for sample radioactivity measurement. The data were decay-corrected, and the biodistribution results were expressed as the percentage injected dose per gram of tissue (%ID/g).

### 2.5. Autoradiography, Western Blotting, and Immunohistochemistry

Following SPECT imaging and biodistribution study, the distal colons of both model and control rabbits were dissected out for autoradiography (ARG) to evaluate tracer uptake. The colon tissue were collected, rinsed, and placed on a super resolution phosphor screen (Perkin-Elmer Cyclone Plus, Perkin-Elmer) for 1 h of exposure. Then, the screen was scanned in a storage phosphor imaging system (Perkin-Elmer Cyclone Plus) to acquire ARG images. Circular ROIs in diameter of 5 mm were set on the image to quantify radioactivity at different regions, and the result was expressed as digital light unit per mm^2^ (DLU/mm^2^).

The colon tissue was also examined by western blotting (WB) and immunohistochemistry (IHC). Briefly, the lysates of fresh colon tissue went through the WB procedure of electrophoresis, transferring to the membrane, and successive incubation with the primary antibody (mouse anti-VCAM-1, Abcam, Cambridge MA, USA) and the secondary antibody (HRP-conjugated goat antimouse IgG, Abcam). The signal bands were acquired to identify VCAM-1 expression in model and control colons, and the common protein glyceraldehyde-3-phosphate dehydrogenase (GAPDH) served as a loading control. To further locate VCAM-1 expression, the IHC study was performed by incubating colon section slices with the primary antibody (mouse anti-VCAM-1, Abcam) and the secondary antibody (HRP-conjugated goat antimouse IgG, Abcam) successively. After the addition of 3,3′-diaminobenzidine substrate, the tissue slices were examined by microscope. The VCAM-1 expression density was determined by image analysis software (Image-Pro Plus, Media Cybernetics Inc., Rockville MD, USA), and the results were expressed as the integrated optical density (IOD).

### 2.6. Statistical Analysis

All measurement data were expressed as the mean ± standard deviation (SD). The results of the model group and the control group were compared using a two-tailed Student's *t*-test. Statistical significance was assumed when *P* < 0.05.

## 3. Results

### 3.1. Characteristics of the Imaging Probe

As described previously [13], the binding affinity of scFv-VCAM-1 to rabbit VCAM-1 antigen was adequate and would not be affected by labeling manipulation. The imaging probe was synthesized by linking ^99m^Tc to scFv-VCAM-1. The fine characteristics of the ^99m^Tc-scFv-VCAM-1 (labeling yield, radiochemical purity, specific activity, and stability) were elucidated in our previous work [13], and kept excellent reproducibility. The cell-binding assay of ^99m^Tc-scFv-VCAM-1 in our other research exhibited a strong affinity to VCAM-1-positive cells [14].

### 3.2. Characteristics of the Colitis Rabbit

**Figure 2:**
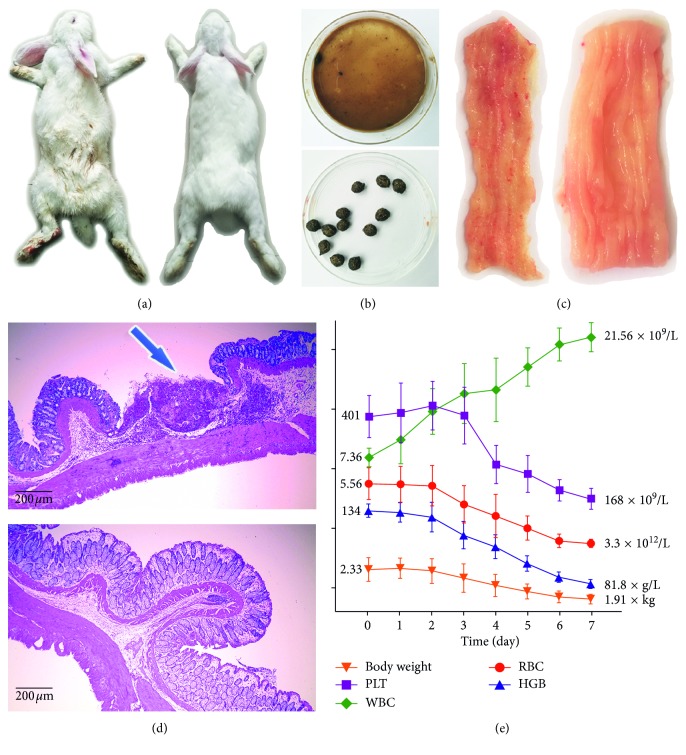
The characteristics of colitis model rabbit. (a) Photos of the model rabbit (left) and the control rabbit (right). The contamination on the foot of the model rabbit indicated diarrhea symptom. (b) Photos of stool in the rectum of the model rabbit (upper) and the control rabbit (lower). Model group stool confirmed diarrhea manifestation. (c) The model colon (left) developed macroscopic colitis with hemorrhagic spots, while the control group (right) kept smooth and intact mucosa. (d) Pictures of model colon (upper) and control colon (lower) HE staining. The blue arrow indicated ulcer and inflammation in the mucous layer of the model colon. (e) The daily changes of model rabbits during 7 days of colitis induction were expressed as trend curves for parameters of body weight, platelet (PLT), white blood cell (WBC), red blood cell (RBC), and hemoglobin (HGB) (mean ± SD, *n*=5).

After the colitis induction with DSS and TNBS, the model rabbits developed typical symptoms of diarrhea and weight loss. Macroscopic colitis with hemorrhagic spots was observed in the model distal colon (Figure 2(c)), and the colon HE staining demonstrated ulcer existence in the mucous layer (Figure 2(d)). In the blood test, the red blood cell (RBC) decreasing and the white blood cell (WBC) increasing confirmed blood loss and inflammation occurrence, respectively, in the model group (Figure 2(e)), whereas the control rabbits presented none of these characteristics. All the results proved the success of inducing colitis in rabbits.

### 3.3. SPECT Imaging

**Figure 3:**
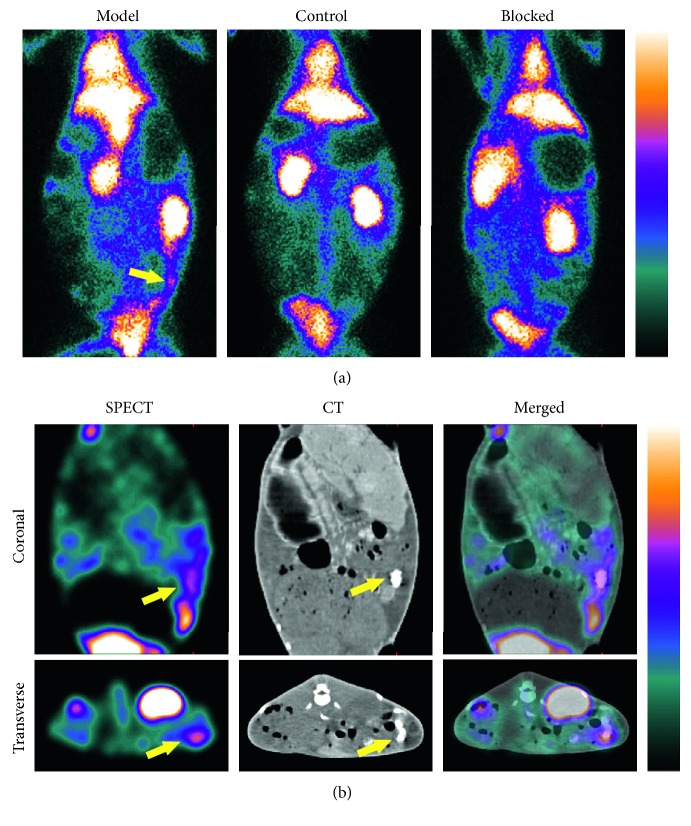
Representative pictures of ^99m^Tc-scFv-VCAM-1 SPECT imaging in rabbits. (a) Probe uptake was observed at the left part of the model rabbit abdomen (yellow arrow) and could be blocked by an excessive unlabeled scFv-VCAM-1. The control group showed weak uptake at the corresponding region. (b) The SPECT/CT images located tracer uptake (yellow arrows) in orthogonal slices. The contrast opacification in CT images indicated distal colon location, where the radioactivity accumulated in SPECT images.

The SPECT planar imaging displayed conspicuous radioactivity accumulation at the left part of the model rabbit abdomen, and it was blocked by a 50-fold dose of excessive unlabeled scFv-VCAM-1 (Figure 3(a)). The control rabbits showed rare foci at the corresponding region. Higher T/NT ratios were observed in the model group (2.71 ± 0.31) than those in the control group (1.12 ± 0.10), which demonstrated statistical significance (*P* < 0.00). The SPECT/CT fusion imaging confirmed the radioactivity distribution of model rabbits in orthogonal slices (Figure 3(b)). The contrast opacification of the iopamidol indicated the tracer accumulation in the distal colon.

### 3.4. Biodistribution Study, Autoradiography, and VCAM-1 Expression Identification

**Figure 4:**
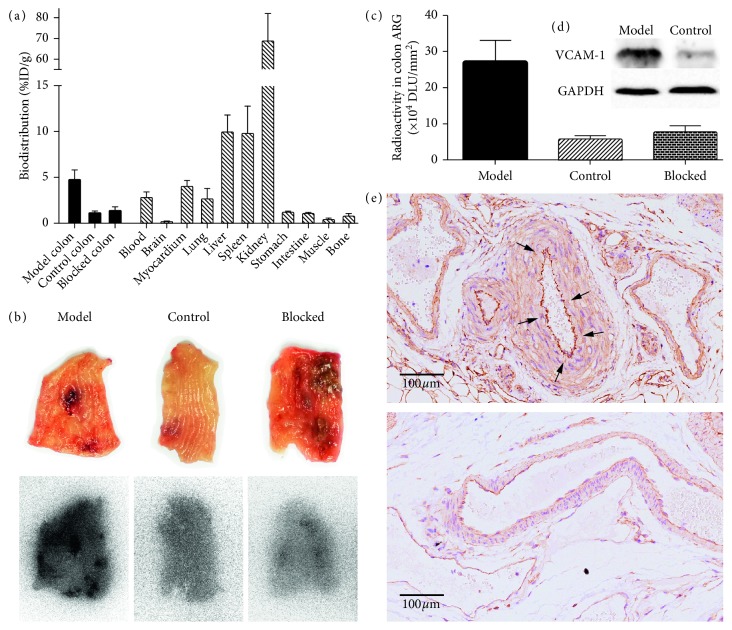
Biodistribution, autoradiography (ARG), and VCAM-1 expression results of the rabbits and colons. (a) The biodistribution of ^99m^Tc-scFv-VCAM-1 in rabbits showed tracer uptake in different tissue and organs after 3 hours of probe administration (mean ± SD, *n*=5). (b) The ARG confirmed tracer uptake in the model colon, with weak accumulation in the control and the blocked colon. (c) Semiquantitative analysis of the ARG region of interest showed more radioactivity in the model group than that in the control and the blocked group (mean ± SD, *n*=10). (d) Western blotting indicated stronger VCAM-1 expression in the model colon than that in the control colon. (e) Immunohistochemistry study demonstrated more VCAM-1 expression in blood vessels of the model colon (upper) than that of the control colon (lower). The strongest VCAM-1 expression (black arrows) located on vascular endothelial cells in the model colon.

The radioactivity accumulation in SPECT imaging was also confirmed by biodistribution in different tissues and organs (Figure 4(a)). The probe uptake in the model colon was higher than that in the control colon. The lesion-to-control ratio was 4.45 ± 1.06, whereas the lesion-to-muscle ratio was 13.67 ± 5.11.

The ARG study confirmed more probe uptake in distal colons of model rabbits than in that of control ones (Figure 4(b)). The radioactivity accumulation could be blocked by excessive unlabeled scFv-VCAM-1. Quantitative analysis of the ARG ROIs (Figure 4(c)) showed that the uptake ratio of the model colon to the control colon was 4.71 ± 0.61. The WB study (Figure 4(d)) of colon tissue indicated stronger VCAM-1 expression in the model group than that in the control group. The IHC study (Figure 4(e)) located VCAM-1 expression on vascular endothelial cells. Quantitative analysis showed that the IOD of VCAM-1 expression in the IHC samples of the model group was 1.65 ± 0.32 times higher than that of the control group.

## 4. Discussion

We used DSS and TNBS to induce colitis in rabbits and used the radioactive imaging probe of ^99m^Tc-scFv-VCAM-1 for the surveillance of colitis lesion. The result of the colon examination and the blood test proved the success of lesion induction. The SPECT imaging demonstrated colitis in both SPECT planar images and SPECT/CT fused images, and the radioactivity accumulation was confirmed by the biodistribution study and ARG. Stronger tracer uptake in ARG and stronger VCAM-1 expression in WB and IHC study were confirmed in the model colon than those in the control colon. These results indicated the feasibility and effectiveness of detecting colitis with the imaging probe of scFv targeting VCAM-1.

Clinical features, such as diarrhea, cramping pain, bloody stool, and weight loss, provide inadequate help for the evaluation of IBD severity, whereas imaging techniques assist a lot in the lesion diagnosis and therapy guidance [4]. As a cost-effective technique with free radiation for IBD, ultrasonography investigates bowel wall thickening and extraintestinal complications such as fistula, stenosis, and phlegmon [16], and is a first-line tool for IBD diagnosis [17]. CT observes bowel wall thickening, mural structure, and haziness of the surrounding mesenteric fat, whereas magnetic resonance imaging (MRI) provides diverse techniques and better parenchyma resolution to visualize pathologic changes and motility alterations of IBD [18]. Endoscopy and biopsies are regarded as the gold standard diagnostic tools [19], but these examinations take along invasive feature and inconvenience. As a noninvasive imaging strategy, molecular imaging can reveal cellular and molecular changes in vivo [20]. The representative molecular imaging techniques utilized in clinical practice are SPECT and positron emission tomography (PET). The common used probes, such as ^18^F-FDG, ^99m^Tc-HMPAO, and ^99m^Tc-IL-8, offer insights into metabolic and functional disorder [21], and can tell the pathological activity and severity in IBD lesion.

Our study used the radioactive probe to visualize colitis lesion in model animal. The *γ*-ray signal has excellent tissue penetration; thus, trace amount of imaging probe is enough to show pathological change. However, the spatial resolution of clinical SPECT is just about 1 cm, so we had to use rabbit as a large-size model animal to investigate colon inflammation. MicroPET keeps outstanding spatial resolution of 1.2 mm, and the imaging results can be expressed semiquantitatively as the standardized uptake value (SUV) [22]. Moreover, due to the absence of lead collimator, microPET has a better sensitivity than SPECT [23]. Our further study may use positron nuclide probe and microPET to detect colitis lesion.

The environment, the genetic make-up, and the gut microbiota are altogether believed to trigger the abnormalities of immune response [24, 25], leading to the chronic inflammation of IBD. In our study, TNBS dissolved in ethanol is administrated to induce colitis lesion imitating IBD pathological feature. The ethanol breaks the mucosal barrier to enable haptenization of TNBS with colonic autologous or microbiota proteins [26], and subsequent transmural cellular infiltration causes impairment of epithelial barrier function, tissue destruction, and perpetuation of inflammation [27]. During this course, VCAM-1 plays an important role in the mediation of leukocyte adhesion and recruitment [28], which makes it feasible to detect colitis lesion in our study. Other studies have used VCAM-1 as an inflammation marker in different organs with different imaging methods, including quantum dot imaging in lungs [29], ^19^F MR nanoparticle spectroscopy in renal [30], microbubble ultrasound imaging in atherosclerosis [31], iron oxide microparticles MR imaging in acute brain inflammation [32], and microPET imaging in tumor therapy evaluation [33]. All these results suggest VCAM-1 as an eligible target for inflammation imaging investigation.

The development of small-sized antibodies facilitates various application. By connecting two variable regions of the immunoglobulin heavy and light chains with a linker peptide, scFv antibody presents much smaller size than intact monoclonal antibody, yet holds the similar specificity [34]. The most significant advantage of scFv as an imaging probe is the fast blood clearance via kidney, which makes it possible to reach a better T/B ratio in a short circulation time. In our imaging study, the probes of scFv-VCAM-1 detected colitis lesion quickly in SPECT scintigraphy. Compared with another VCAM-1 monoclonal antibody imaging study [8], our imaging takes shorter time (3 h vs. 4 h) and achieves a better T/B ratio (2.7 vs. 2.2). However, we notice that the radioactivity accumulation in the colon lesion is not as strong as we expected. Although the lesion-to-muscle ratio is 13.67 in biodistribution study, in ARG study, the uptake ratio of the model colon to the control colon is just 4.71. We believe that some details may lead to this result. First, scFv has a common problem of weaker binding affinity than monoclonal antibody [35], so specific probe accumulation in colitis lesion is not strong enough. Second, according to our biodistribution study, there is more tracer uptake in the control intestine and colon than that in the muscle. This explains lower ratio of lesion-to-control than that of lesion-to-muscle. Third, the WB and IHC study shows moderate VCAM-1 expression in the model colon, with 1.65 times higher IOD in the IHC study of the model group than that of the control group, which also causes suboptimal contrast in SPECT imaging. Therefore, the imaging performance of scFv-VCAM-1 probe needs further improvement.

We would like to elucidate the limitations of our study. First, the colon lesion of the model animal may not represent the real biological status of IBD. It takes 7 days to induce colon lesion with DSS and TNBS; thus, the inflammation in site is an acute response. The pathological feature in the lesion is more like ulcerative colitis than Crohn's disease. The characteristics of the SAMP1/YitFc mouse model have many similarities to those of Crohn's disease [36], which may be an eligible IBD model animal. Second, further investigation is needed to evaluate the imaging performance of scFv-VCAM-1 probe. Although the probe successfully detects colon lesion in SPECT imaging, many factors are making influence to the result, such as probe-binding affinity, lesion blood perfusion, and probe pharmacokinetics. Moreover, the enhanced permeability and retention effect may also affect probe accumulation in the lesion; thus, we need irrelevant scFv for none-specific imaging investigation. Our future work will focus on these aspects to improve the imaging performance.

## 5. Conclusion

We prepared colitis model rabbit with DSS and TNBS, and used the animal for the investigation of ^99m^Tc-scFv-VCAM-1 imaging probe. The SPECT imaging, the biodistribution, and the ARG results confirmed the probe's ability to detect colitis lesion. Our study indicated the promising way of evaluating IBD lesion with the imaging probe of scFv targeting VCAM-1.

## Data Availability

The images and their analysis results data used to support the findings of this study are included within the article.
